# Use of concurrent multiple methods of contraception in the United States, 2008 to 2015

**DOI:** 10.1016/j.conx.2021.100060

**Published:** 2021-02-08

**Authors:** Megan L. Kavanaugh, Emma Pliskin, Jenna Jerman

**Affiliations:** a Guttmacher Institute in New York, New York, NY, United States; b Formerly at the Guttmacher Institute in New York, New York, NY, United States

**Keywords:** Condoms, Contraceptive use, Dual use, National Survey of Family Growth, United States

## Abstract

**Objective:**

To identify prevalence of, characteristics associated with, and combinations of, use of more than one method of contraception at last intercourse among US women between 2008 and 2015.

**Methods:**

We conducted bivariate and multivariable logistic regression analyses using data on concurrent contraceptive use from 2 nationally representative samples of women ages 15 to 44 who had used some form of contraception at last intercourse in the past 3 months in the 2006–2010 (*n* = 6601) and 2013–2017 (*n* = 5562) cycles of the National Survey of Family Growth.

**Results:**

Use of more than one method of contraception at last sex increased from 14% in 2008 to 18% in 2015 (*p*<0.001), with increases in use documented across many population groups. Among multiple method users, the majority combined condoms with other methods (58%), while the rest combined other methods (42%). When compared to single method users, dual method users employing condoms are a more homogeneous group of individuals than are dual method users not employing condoms. As age increases, dual use with condoms decreases, but there is no similar linear relationship between age and dual method use without condoms.

**Conclusions:**

A sizable proportion of US women use more than one contraceptive method during sex; current estimates of contraceptive use focused exclusively on single method use may underestimate the extent to which women are protected from unintended pregnancy. The needs and goals of individuals combining contraceptive methods in different ways may change over the life course as pregnancy desires and life circumstances change.

**Implications:**

A sizable proportion of US women use more than one contraceptive method during sex; clinicians and health educators in nonclinical settings should assess and acknowledge these more complicated contraceptive strategies in order to help individuals achieve autonomy in method choice and meet their goals around pregnancy and sexually transmitted infection prevention.

## 1 Introduction

Use of contraception confers substantial benefits to individuals who desire to delay, space, or prevent pregnancies. The United States has a range of contraceptive options available; however, as of the most recent national data, most women who use contraception continue to rely on 4 methods as their primary form of contraception: tubal ligation or tubal implants (22%); the pill (22%), condoms (15%), and IUDs (14%) [1]. These widely cited contraceptive use statistics, which come from the National Survey of Family Growth (NSFG), rely on a contraceptive effectiveness hierarchy to reflect a single primary method in situations where women report more than one method, simplifying contraceptive use patterns and hiding multiple method use [2,3].

Other evidence from the NSFG, considered to be the most comprehensive national-level information about individuals’ sexual and reproductive health, indicates that women often employ more than one method of contraception, whether sequentially or concurrently. In 2006–2010, approximately 9% of all female contraceptive users reported use of more than one method during the past month [2], and in 2013–2015 about 17% reported this practice [2,4]. These reports of multiple method use from the NSFG represent any method use during the month prior to interview, making it difficult to assess from these survey items whether methods reported were used at the same act of intercourse (concurrent use) or at different acts within the prior month. Accurately assessing concurrent method use from these survey items is especially a challenge for those individuals who may have used multiple coital-specific methods, such as condoms and withdrawal, in the past month.

Other work has shown that the actual level of concurrent use of methods may be higher than documented in the NSFG, which may have to do with the ways in which method use questions are asked in a questionnaire. In a 2012 national study of women recruited through an online panel, in which they were asked separate questions about use of hormonal and coital methods and listed withdrawal first in the latter question (a different approach to querying about contraceptive use than is used in the NSFG), approximately one-third of women at risk of unintended pregnancy reported using more than one method during the previous 4 weeks. These respondents used methods either concurrently or switched between methods during the time period, with 39% of users of highly effective methods also reporting using condoms, withdrawal or both [5]. A 2009 nationally representative study of 18 to 44 year olds documented dual use of highly effective methods with condoms among 12% of male and female respondents in 2009 [6]. Qualitative research has shed light on the variety of strategies that contraceptive users employ when combining methods, specifically by backing up inconsistent method use with other methods and by “buttressing,” or reinforcing, methods [7].

Much of the work around concurrent method use to date has focused on dual use of a prescription contraceptive method with condoms [8], [9], [10], [11]. Given the complementary benefits of pregnancy prevention with prevention of sexually transmitted infections (STIs), this combination is a logical one. However, users may combine methods for many reasons, and documenting multiple method use beyond use that includes condoms remains an important task in filling out the picture of contraceptive use in the United States. Use of more than one method simultaneously may also have implications for failure rates of any individual contraceptive method, especially in light of recent improvements in failure rates for the most commonly used contraceptive methods in the United States [12]. Our objectives with this analysis were to identify the prevalence of, characteristics associated with, and combinations of, concurrent contraceptive use at last intercourse, with specific attention to differences between multiple method users employing condoms and those not employing condoms,.

## 2 Methods

Data for this cross-sectional, descriptive study come from the female respondent files of the 2006–2010 and 2013–2017 National Surveys of Family Growth (NSFG).^1^ The NSFG uses a multistage probability sampling design that oversamples Black and Hispanic groups and adolescents ages 15 to 19. These in-home, face-to-face interviews of US residents aged 15 to 44 in the household population of the United States provide the most comprehensive nationally representative information available on contraceptive use in the United States. More detailed information on survey methodology, sample design, response rates, fieldwork procedures, and variance estimation is published elsewhere (https://www.cdc.gov/nchs/data/nsfg/NSFG_2013–2015_Summary_Design_Data_Collection.pdf, and the data are deidentified and publicly available for download on the NSFG website (http://www.cdc.gov/nchs/nsfg.htm). Given the deidentified nature of the public use data in the data set, our organization's institutional review board (Department of Health and Human Services identifier IRB00002197) determined that this analysis was exempt from institutional review board approval.

For simplicity, we present results for the years 2008 and 2015, representing the midpoint of each year of data collection. The samples consist of 6601 (2006–2010) and 5562 (2013–2017) females ages 15 to 44^2^ who were sexually active (penile-vaginal intercourse) in the 3 months prior to interview and reported some form of contraceptive use at last sex. Women who reported using sterilization, or use of permanent contraception, are included in this analysis because method use can be motivated by multiple factors beyond solely pregnancy prevention [14].

The primary outcome of interest in this study is concurrent multiple method use, as measured at last sex within the past 3 months, based on a list of possible methods presented to female NSFG respondents and captured by the variables METH3M1, METH3M2, METH3M3, and METH3M4 (in contrast to the CONSTAT variables more commonly used to capture contraceptive use during the month prior to interview). These METH3M variables represent the first, second, third, and fourth method the respondent indicated using at the same, most recent, act of intercourse. Participants were considered to be multiple method users if they reported using at least a second method. Independent variables include demographic and sexual and reproductive health characteristics that may be associated with contraceptive method use. Characteristics include age, race and ethnicity, income as a percentage of the federal poverty level, nativity, relationship status, education, health insurance coverage, parity and number of future births expected. Additional characteristics available only for 2013–2017 are lifetime sexual experience with a same sex partner, sex with a non-monogamous partner in the past year, and receipt of an STI test or treatment in the past year. The latter two characteristics serve as proxies for STI risk, which may influence condom use.

To examine change in the use of concurrent multiple methods over time, we pooled the 2006–2010 and 2013–2017 female respondent files and weighted each time period accordingly, using the midpoints to represent each (2008 and 2015, respectively). We then used bivariate logistic regression to test for significant differences in the dependent variable (concurrent multiple contraceptive method use) between the two time periods overall (one model) and by population subgroups (12 models, one for each sociodemographic characteristic examined). We highlight findings significant at *p* <= 0.01 in the text. For the most recent time period, we further examined which methods were being combined among users of at least two methods at last intercourse. In presenting method dyads, we developed a hierarchy for grouping methods, with condoms, withdrawal and permanent methods being prioritized for consideration in each method dyad in that order. For example, if a user reported using both condoms and withdrawal, they were grouped in the category of “condom and another method” and not in the “withdrawal and another method” category.

Given the prevalence of combining condoms with another method at last intercourse and the research interest in this practice, we examined factors associated with dual use including condoms and dual use excluding condoms in the 2013–2017 time period. Among sexually active female respondents who used at least one method at last sex, we constructed a categorical variable to represent 3 distinct concurrent method use strategies: single method use (referent), dual method use including a condom, and dual method use not including a condom. We used multinomial logistic regression to determine individual characteristics associated with each type of method use, and to compare these characteristics across types of method user. All demographic and sexual and reproductive health characteristics examined at the bivariate level were initially included in the multinomial model but were removed if they were not significantly associated with any of the outcome categories at *p* < 0.1. We included age, race/ethnicity and poverty status in the model regardless of significance due to their theoretical relevance to method use.

All analyses were conducted using the “svy” command prefix within Stata 15.1 to account for the NSFG's use of a multistage probability sample.

## 3 Results

Use of more than one method of contraception at last sex among all sexually active women who use contraception increased significantly from 14% in 2008 to 18% in 2015, with increases in use documented across many population groups (Table 1). In particular, respondents who identified as non-Hispanic white, had an income of 200% to 299% of the federal poverty level, were U.S. born, were not married or cohabiting, had some college education, had private insurance coverage, were nulliparous, and who expected 0 to 2 future births all used multiple methods of contraception at last sex in 2015 at a higher rate than respondents in these categories in 2008 (*p* <= 0.01).

**Table 1:** Characteristics of sexually active women ages 15–44 using at least two methods at last sex in the past three months in the NSFG between 2008 (*N* = 6601) and 2015 (*N* = 5562)

	2008 (*N* = 6601)	2015 (*N* = 5562)	
	%	%	*p*-value
Overall	13.8%	17.8%	< 0.001
N	6601	5562	
Age			
15–19 y	31.5%	41.2%	0.03
20–24 y	23.2%	29.4%	0.05
25–29 y	12.7%	17.2%	0.03
30–34 y	11.4%	12.5%	0.62
35–39 y	9.0%	10.1%	0.56
40–44 y	6.9%	11.9%	0.01
Race/Ethnicity			
White, NH	14.5%	19.5%	0.00
Black, NH	15.4%	19.3%	0.07
Other/Multiple, NH	14.6%	15.6%	0.78
Hispanic	9.7%	13.3%	0.05
Federal poverty level			
0–99%	15.3%	19.0%	0.07
100–199%	15.1%	16.7%	0.49
200–299%	12.3%	19.1%	0.00
300%+	13.2%	17.1%	0.03
Nativity status			
US born	14.8%	19.0%	*p* < 0.001
Foreign born	8.7%	11.8%	0.16
Relationship status			
Married	7.1%	9.1%	0.11
Cohabitating	12.9%	14.0%	0.63
Not married or cohabitating	25.3%	32.5%	0.00
Educational attainment			
Not HS grad	13.1%	19.6%	0.01
HS grad/GED	13.1%	13.9%	0.72
Some college	15.6%	21.7%	0.00
College grad	13.0%	16.0%	0.10
Current insurance coverage			
Private	14.7%	18.5%	0.01
Medicaid	15.5%	15.7%	0.95
Other public	13.0%	25.0%	0.04
None	10.2%	15.5%	0.02
Parity			
0	22.8%	29.0%	0.01
1–2	9.5%	12.4%	0.04
3 or more	9.1%	10.4%	0.46
Number of future births expected			
0	9.5%	12.6%	0.01
1–2	17.4%	22.7%	0.01
3 or more	28.6%	32.1%	0.45
Any same sex sexual contact			
Never had same sex sexual contact	NA	17.4%	
Has had same sex sexual contact	NA	20.1%	
Received STI test or treatment^a^			
No STI test or treatment	NA	14.5%	
Received STI test or treatment	NA	24.2%	
Had sex with non-monogamous partner^a^			
No sex with non-monogamous partner	NA	16.7%	
Had sex with non-monogamous partner	NA	27.0%	

Notes: Population includes female respondents who had sex in the past 3 months, used at least one contraceptive method at last sex, and were 15–44 years old. Survey years in column headings represent the midpoint of data collection years for each of the 2 time periods covered by the NSFG surveys. Data are all%. Population weighted to reflect the US female civilian population of the United States. Logistic regression models were used to test for significant differences in the proportion of dual method users between time periods.

NA, not applicable.

a In the 12 months prior to interview.

Among women who used contraception at last sex in the past three months in 2015, 18% reported use of *at least*2 methods and 2% reported use of 3 or more methods (not shown). In 2015, there were 53 unique dyad combinations of methods at last sex among multiple method users (not shown). Among those multiple method users, the majority combined condoms with other methods (58%), while the rest did not include condoms among their method combinations (42%) (Figure). Specifically, the most common method combinations among dual users of contraception in 2015 were condoms and short-acting reversible contraceptive methods (31%), withdrawal and short-acting reversible contraceptive methods (16%), condoms and withdrawal (10%), withdrawal and other methods besides condoms or short-acting reversible methods (7%) and condoms and long-acting reversible contraceptive methods (7%) (not shown).

**Figure:**
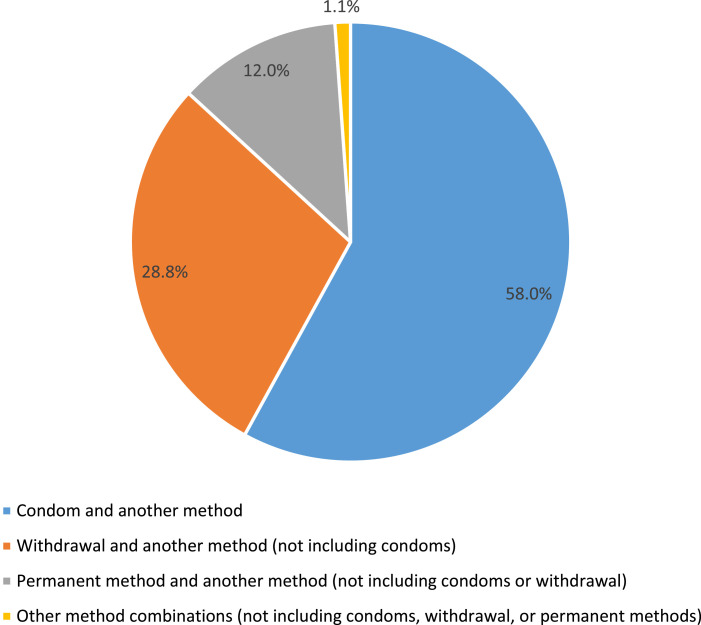
Contraceptive method dyads among dual contraceptive users at last sex in past 3 months, 2015 (*N* = 925). Notes: Population includes female contraceptive users who used at least two methods at last sex including permanent methods (e.g., tubal ligation, partner's vasectomy). Population weighted to reflect the US female civilian population of the United States. Within method dyads, condoms, withdrawal and permanent methods are prioritized for consideration in that order (e.g., if a user reported using both condoms and withdrawal, they were grouped in the category of “condom and another method” and not in the “withdrawal and another method” category).

Results from multinomial multivariable models suggest that user characteristics associated with concurrent dual contraceptive use involving condoms differed from those associated with dual use without condoms, when comparing both to single method users (Table 2). Among contraceptive users, 82.2% used one method, 10.3% used condoms combined with another method, and 7.5% used 2 methods that did not include condoms. After adjusting for key demographic and sexual and reproductive health characteristics, in comparison with single method users, nonmarried women (both cohabiting and not), and those who had received tests or treatments for STIs in the past year were more likely to be dual method condom users while women older than age 19, Hispanic and non-Hispanic women of multiple or other races, those with Medicaid health insurance, and those who had ever had a same sex sexual experience were all less likely to be dual method condom users. In contrast, only those women who were neither married nor cohabiting and those who reported some college education were more likely to be noncondom dual method users, while those who identified as non-Hispanic Black or Hispanic and those who had sex with a nonmonogamous partner, were less likely to be noncondom dual method users as compared to single method users.

**Table 2:** Percentage distributions of dual method contraceptive use with and without condoms, and adjusted relative risk ratios and confidence intervals of dual method users including and excluding condoms compared to single method users, among all sexually active women ages 15–44 using a contraceptive method at last sex in the past three months in the NSFG, 2015 (*N* = 4179)

	Dual method condom users vs Single method users				Dual method users without condoms vs Single method users			
	Percent	RRR	Confidence Intervals		Percent	RRR	Confidence Intervals	
Overall	10.3%				7.5%			
Age								
15–19 y	32.3%				9.0%			
20–24 y	17.5%	0.59	0.36	0.98^b^	11.8%	1.34	0.66	2.72
25–29 y	10.8%	0.48	0.30	0.76^c^	6.3%	0.69	0.36	1.30
30–34 y	6.2%	0.34	0.19	0.63^d^	6.3%	0.72	0.30	1.72
35–39 y	5.5%	0.38	0.20	0.75^c^	4.7%	0.55	0.25	1.20
40–44 y	3.7%	0.25	0.12	0.52^d^	8.2%	0.96	0.42	2.21
Race								
White	10.9%				8.6%			
Black, NH	15.1%	0.84	0.59	1.21	4.2%	0.43	0.27	0.68^d^
Other/Multiple, NH	5.0%	0.40	0.26	0.62^d^	10.6%	1.07	0.63	1.81
Hispanic	8.7%	0.65	0.44	0.96^b^	4.6%	0.48	0.28	0.83^c^
Poverty								
0–99%	11.5%				7.5%			
100–199%	10.5%	0.86	0.54	1.35	6.3%	0.59	0.34	1.03
200–299%	10.8%	0.89	0.58	1.35	8.3%	0.75	0.37	1.53
300%+	9.3%	0.80	0.53	1.21	7.8%	0.65	0.35	1.23
Relationship status								
Married	2.7%				6.3%			
Cohabitating	6.7%	1.89	1.24	2.90^c^	7.3%	1.14	0.68	1.92
Not married or cohabitating	23.2%	6.73	4.36	10.38^d^	9.2%	1.89	1.18	3.03^c^
Educational attainment								
Not HS grad	13.5%				6.1%			
HS grad/GED	8.4%	0.78	0.50	1.24	5.4%	0.89	0.48	1.67
Some college	12.0%	1.08	0.73	1.62	9.7%	1.69	1.01	2.82^b^
College grad	8.8%	1.27	0.69	2.32	7.2%	1.31	0.72	2.38
Current insurance coverage								
Private	10.6%				7.9%			
Medicaid	10.3%	0.66	0.45	0.96^b^	5.4%	0.50	0.23	1.08
Other public	12.4%	1.19	0.62	2.27	12.6%	1.73	0.67	4.50
None	8.7%	0.79	0.51	1.22	6.8%	0.92	0.48	1.76
Parity								
None	19.3%				9.7%			
1–2	6.1%	0.73	0.51	1.02	6.3%	0.95	0.59	1.51
3+	4.1%	0.62	0.38	1.01	6.3%	1.08	0.61	1.90
Ever same sex sexual experience								
Never	10.4%				6.9%			
Has experienced	9.9%	0.65	0.46	0.91^b^	10.2%	1.47	0.95	2.29
Had sex with non-monogamous partner^a^								
No sex with non-monogamous partner	9.2%				7.6%			
Had sex with non-monogamous partner	21.2%	1.02	0.72	1.45	5.8%	0.50	0.29	0.86^b^
Received tests or treatment for STDs^a^								
No STD test/treatment	7.2%				7.2%			
Received STD test/treatment	16.2%	1.58	1.13	2.19^c^	8.0%	1.19	0.82	1.73

Notes: Population includes female respondents who had sex in the past 3 months and were 15–44 years old. Population weighted to reflect the US female civilian population of the United States. Multinomial logistic regression models were used to test for significant differences in the proportion of dual method use with and without condoms by user characteristics to single method use by user characteristics.

a In the 12 months prior to interview.

b Significant difference between years at *p* < 0.05 based on multinomial logistic regression analyses.

c Significant difference between years at *p* < 0.01 based on multinomial logistic regression analyses.

d Significant difference between years at *p* < 0.001 based on multinomial logistic regression analyses.

## 4 Discussion

Almost 1 in 5 contraceptive users employed more than one method at last sex, and the proportion who did so increased 4 percentage points between 2008 and 2015. This small but significant increase in recent years has implications for our understanding of women's pregnancy prevention strategies. Notably, contraceptive use at the national level as measured by the single, most effective method used did not change significantly during this same time period [13] and was more common among users ages 20 and older than those younger than 20 during this same time period [15]. Reflecting some progress towards a specific goal outlined in the Healthy People 2020 federal initiative, concurrent multiple method use is most common among young women ages 15 to 19, and has become more so between the time periods examined [16]. Use of more than one method at last sex also decreases steadily with age. Younger age and other characteristics often linked with young age, such as not being married or living with a partner, also represent individual characteristics historically associated with both having higher rates of unintended pregnancy [17] and higher rates of STIs [18]. As such, it may be that individuals who fall into these population groups have stronger desires to avoid pregnancy [19] and/or recognize their increased risk of STIs [20] so use one or more methods to protect themselves against pregnancy and, when using condoms as one of the methods, STIs. In addition, unlike single, most effective contraceptive method use at the national level, which differs by race/ethnicity such that non-Hispanic Black women have slightly lower levels of overall use by this metric as compared to non-Hispanic white women, levels of concurrent multiple method use in our analysis are similar between Black and white women. Further research into whether white women and Black women employ contraceptive strategies in different ways and for different reasons could help to shed light on these observed differences in single versus multiple method use.

The sheer number of unique combinations of methods provides evidence that women's motivations for multiple method use are complex, suggesting that the measurement and interpretation of these practices must be further refined. People have a variety of reasons for selecting a particular method or methods of contraception [14], and ensuring that measures accurately reflect contraceptive preferences and behaviors is critical. Measures that only assess concurrency of method use at a single sexual encounter instead of over time insufficiently capture the various ways in which contraceptive users combine methods to engage in pregnancy and STI prevention.

Dual method use has traditionally been defined as the use of condoms with another contraceptive method, and the majority of research on multiple method use has focused on this dyad. While dual use with condoms still represents the majority of overall multiple method use, the findings of this study indicate that many women combine methods other than condoms, suggesting that multiple method use may be part of more complicated and nuanced pregnancy prevention strategies beyond the layering of STI prevention with pregnancy prevention. This supports previous research demonstrating that concurrent multiple method use may be motivated by many factors, including sexual pleasure, partner preferences, and a desire to increase the perceived level of protection from pregnancy from one method alone [7].

When compared to single method users, dual method users employing condoms are a more homogeneous group of individuals than are dual method users not employing condoms. As age increases, dual use with condoms decreases, but there is no similar linear relationship between age and dual method use without condoms. This finding suggests that the needs and goals of those combining methods in different ways may change over the life course as fertility desires and life circumstances change. For example, young people may be more likely to have multiple sexual partners [10], which may drive a desire for STI prevention alongside pregnancy prevention. Our findings of a higher likelihood of dual use with condoms among individuals who were not married or cohabiting and among those who had received STI tests or treatment support this theory. Users who combine methods not including condoms, on the other hand, are a much more heterogeneous group, with fewer differences in this practice by demographic characteristics. These differences between the types of dual method users underscore the importance of tailored health education messages during clinical and nonclinical encounters regarding contraceptive method options; at the very least, clinicians and educators should be discussing both STI prevention and additional layers of pregnancy prevention as factors to consider when making contraceptive choices. Future research should explore the differing motivations for combining different methods, especially among multiple method users that do not include condoms in their mix.

Acknowledging the prevalence of multiple method use among contraceptive users in the United States also has implications for typical use failure rates associated with each individual method which, in turn, can impact how clinicians provide information to patients about the range of method options available. Recent studies indicate that these failure rates have largely been declining in recent years, given increases in the use of highly effective methods and shifts in the demographics of method users [12]. However, because method-specific failure rates are calculated based on the protection offered by the most effective method used during a given timeframe, failure rates for less effective methods such as condoms and withdrawal, which are often combined with more effective methods, may be somewhat imprecise. Thus, future efforts to calculate typical use failure rates for methods should take common contraceptive dyads into consideration.

This study is limited by its cross-sectional design and the inherent limitations of contraceptive method use measurement. Data are based on self-reports of contraceptive method used at last sex in the past 3 months, allowing for multiple mentions of methods. However, it is possible that respondents underreported some methods used at last sex due to recall failure or because they did not mention reinforcing strategies, such as withdrawal or periodic abstinence, in combination with a hormonal method. Further, the limited 3-month period may not fully represent how contraceptive users alter their method use and combination strategies over time based on changing life circumstances. Relying on a single item that queries about all method use rather than splitting items to ask about hormonal method use separate from coital-specific method use may underestimate the latter type of use [5], and thus overall concurrent method use. Finally, the documented increase in multiple contraceptive use at last sex between the time periods may be influenced by increased reporting of a broader range of contraceptive methods, including withdrawal, and a normalization of withdrawal as a viable contraceptive method option that has increased between the time periods [5].

A substantial minority of contraceptive users employs more than one method of contraception at the same act of intercourse, and this proportion has increased in recent years. Motivations beyond “doubling up” on both pregnancy and STI prevention undergird multiple method use, as evidenced by the proportion of multiple method users that do not use condoms. Clinicians and health educators in nonclinical settings should assess and acknowledge these more complicated contraceptive strategies during educational encounters in order to help individuals achieve autonomy in method choice and ensure they are able to access and use the method or methods that best suit their needs. As our understanding of how contraceptive users and their partners employ different strategies to protect against either/both pregnancy and, sometimes, STIs continues to evolve, researchers must continue to interrogate the extent to which existing metrics of contraceptive use accurately reflect contraceptive users’ realities. Acknowledging the use of multiple methods in our ongoing surveillance of contraceptive use is one step in this direction.

## Footnotes

1 All NSFG respondents self-report their current gender at the time of interview, which determines the questionnaire they are routed into. For this analysis, we use the terms “women” and “female” to mirror NSFG language, but not all respondents included in the female NSFG respondent file may identify in this way.

2 The 2015-2017 NSFG included an additional 668 female respondents aged 45-49 at the time of screening; these individuals were excluded from all analyses as respondents from this age group were not included in the 2006-2015 data.
